# Associations between Blood and Urine Arsenic Concentrations and Global Levels of Post-Translational Histone Modifications in Bangladeshi Men and Women

**DOI:** 10.1289/ehp.1510412

**Published:** 2016-03-11

**Authors:** Caitlin G. Howe, Xinhua Liu, Megan N. Hall, Vesna Slavkovich, Vesna Ilievski, Faruque Parvez, Abu B. Siddique, Hasan Shahriar, Mohammad N. Uddin, Tariqul Islam, Joseph H. Graziano, Max Costa, Mary V. Gamble

**Affiliations:** 1 Department of Environmental Health Sciences,; 2 Department of Biostatistics, and; 3 Department of Epidemiology, Mailman School of Public Health, Columbia University, New York, New York, USA; 4 Columbia University Arsenic Project in Bangladesh, Dhaka, Bangladesh; 5 Department of Environmental Medicine, Langone Medical Center, New York University, New York, New York, USA

## Abstract

**Background:**

Exposure to inorganic arsenic is associated with numerous adverse health outcomes, with susceptibility differing by sex. Although evidence from in vitro studies suggests that arsenic alters post-translational histone modifications (PTHMs), evidence in humans is limited.

**Objectives:**

The objectives were to determine: a) if arsenic exposure is associated with global (percent) levels of PTHMs H3K36me2, H3K36me3, and H3K79me2 in a sex-dependent manner, and b) if %PTHMs are stable when arsenic exposure is reduced.

**Methods:**

We examined associations between arsenic, measured in blood and urine, and %PTHMs in peripheral blood mononuclear cells from 317 participants enrolled in the Bangladesh Folic Acid and Creatine Trial (FACT). We also examined the stability of %PTHMs after the use of arsenic-removal water filters (n = 60).

**Results:**

Associations between natural log–transformed (ln) urinary arsenic, adjusted for creatinine (uAsCr), and %H3K36me2 differed significantly between men and women (p = 0.01). ln(uAsCr) was positively associated with %H3K36me2 in men [β = 0.12; 95% confidence interval (CI): 0.01, 0.23, p = 0.03] but was negatively associated with %H3K36me2 in women (β = –0.05; 95% CI: –0.12, 0.02, p = 0.19). The patterns of associations with blood arsenic were similar. On average, water filter use was also associated with reductions in %H3K36me2 (p < 0.01), but this did not differ significantly by sex. Arsenic was not significantly associated with %H3K36me3 or %H3K79me2 in men or women.

**Conclusions:**

Arsenic exposure was associated with %H3K36me2 in a sex-specific manner but was not associated with %H3K36me3 or %H3K79me2. Additional studies are needed to assess changes in %H3K36me2 after arsenic removal.

**Citation:**

Howe CG, Liu X, Hall MN, Slavkovich V, Ilievski V, Parvez F, Siddique AB, Shahriar H, Uddin MN, Islam T, Graziano JH, Costa M, Gamble MV. 2016. Associations between blood and urine arsenic concentrations and global levels of post-translational histone modifications in Bangladeshi men and women. Environ Health Perspect 124:1234–1240; http://dx.doi.org/10.1289/ehp.1510412

## Introduction

Throughout the world, > 140 million people are exposed to arsenic-contaminated drinking water (Bagchi 2007); in Bangladesh alone, ≤ 57 million individuals are exposed (Kinniburgh et al. 2003). Chronic exposure to arsenic causes bladder, lung, and skin cancers and is also associated with numerous noncancer health outcomes [National Research Council (NRC) 2013]. Susceptibility to many of these arsenic-related health outcomes differs by sex, with some outcomes preferentially afflicting males and others females (NRC 2013). For example, males are more likely to develop arsenic-induced skin lesions (Ahsan et al. 2006b; Watanabe et al. 2001) and skin, liver, and bladder cancers (Chen and Wang 1990; Chen et al. 2003; Leonardi et al. 2012), whereas females may be more susceptible to arsenic-induced developmental outcomes (Gardner et al. 2013; Hamadani et al. 2011; Saha et al. 2012) and cardiovascular disease (CVD) (Moon et al. 2013). However, the mechanisms underlying these sex differences remain unknown.

Experimental studies and observational studies in human populations have demonstrated that arsenic alters epigenetic modifications, including global 5-methylcytosine (5-mC) (Niedzwiecki et al. 2013; Pilsner et al. 2007; Ren et al. 2011; Tellez-Plaza et al. 2014) and 5-hydroxymethylcytosine (5-hmC) (Niedzwiecki et al. 2015; Zhang J et al. 2014), and there is evidence that these effects differ by sex (Broberg et al. 2014; Niedzwiecki et al. 2015; Nohara et al. 2011; Pilsner et al. 2012). *In vitro* and rodent studies have also shown that arsenic alters global (percent) post-translational histone modifications (PTHMs) in tissues or in cell lines derived from tissues that are targets of arsenic toxicity, such as the lung (Zhou et al. 2008), bladder (Chu et al. 2011), and brain (Cronican et al. 2013), and the effects of arsenic on %PTHMs in the brain have been shown to be sex-dependent in mice (Tyler et al. 2015). An epidemiological study of 63 male steel workers reported that arsenic exposure via inhalation was associated with increased global levels of histone H3 lysine 4 dimethylation in white blood cells (WBCs) (Cantone et al. 2011); however, because this study only included men, potential differences by sex could not be examined.

In a previous study of 40 Bangladeshi adults, we observed sex-specific associations between arsenic, measured in urine, and several %PTHMs (H3 lysine 4 trimethylation, H3 lysine 27 trimethylation, and H3 lysine 27 acetylation) in peripheral blood mononuclear cells (PBMCs) (Chervona et al. 2012). We have also previously observed sex-specific associations between arsenic exposure and global levels of both 5-mC and 5-hmC in PBMC DNA (Niedzwiecki et al. 2015). Thus, we now present data on three PTHMs: histone H3 lysine 79 dimethylation (H3K79me2), selected because this modification has been shown to regulate the expression of Tet1 (Huang et al. 2013; Williams et al. 2011), which converts 5-mC to 5-hmC (Ito et al. 2011), and because it is dysregulated in cancers (Bernt et al. 2011; Zhang L et al. 2014), and histone H3 lysine 36 di- and trimethylation (H3K36me2 and H3K36me3, respectively), selected because these two PTHMs have been shown to be altered by arsenic *in vitro* (Zhou et al. 2008) and because they are also dysregulated in several types of cancer (Duns et al. 2010; Fontebasso et al. 2013; He et al. 2011; Tamagawa et al. 2013). This study utilized a subset of PBMC samples collected from participants enrolled in the Folic Acid and Creatine Trial (FACT) (Peters et al. 2015). First, we evaluated sex-specific associations between arsenic and our candidate PTHMs using baseline FACT samples. Then, because all participants in the trial were provided with arsenic-removal water filters at enrollment, we evaluated whether %PTHMs were altered after reduction of arsenic exposure; this evaluation was performed using samples collected at baseline and at week 12 from participants who did not receive a dietary supplement. The data reported herein add to a growing body of evidence indicating that arsenic induces epigenetic dysregulation on a global level and, moreover, that this dysregulation often occurs in a sex-specific manner.

## Methods

### Region and Participants

In 2010, participants for the present study (FACT) were recruited from the Health Effects of Arsenic Longitudinal Study (HEALS), a prospective cohort study that initially recruited 11,746 adults (between the ages of 20 and 65) living in a 25 km^2^ region in Araihazar, Bangladesh (Ahsan et al. 2006a). FACT participants were randomly selected from all HEALS participants who had been drinking from household wells with water arsenic ≥ 50 μg/L, the Bangladesh standard for safe drinking water. Exclusion criteria included pregnancy, nutritional supplement use, and adverse health outcomes, including proteinuria, renal disease, diabetes, gastrointestinal disease, chronic obstructive pulmonary disease, skin lesions, and cancer. Written informed consent was obtained by Bangladeshi field staff physicians. This study was approved by the Institutional Review Board of Columbia University Medical Center and the Bangladesh Medical Research Council.

### Study Design

The FACT study is a randomized, placebo-controlled trial that had the primary goal of determining whether folic acid (FA) and/or creatine supplementation reduces blood arsenic (bAs) concentrations in arsenic-exposed Bangladeshi adults (Peters et al. 2015). All FACT participants received an arsenic-removal water filter (READ-F filter, Brota Services International) at baseline to be used for the duration of the study and thereafter. Participants (*n* = 622) were also randomized to one of five nutrition intervention treatment arms: placebo (*n* = 104), 400 μg FA/day (*n* = 156), 800 μg FA/day (*n* = 154), 3 g creatine/day (*n* = 104), and 3 g creatine + 400 μg FA/day (*n* = 104) (Peters et al. 2015). Whole blood and urine samples were collected from participants at baseline, week 12, and week 24; sample collection and handling have been described previously (Chervona et al. 2012; Peters et al. 2015). For the present study, we used histones isolated from PBMCs collected at baseline (i.e., pre-intervention) from a subset of FACT participants from all five treatment arms (see Figure S1), who had whole blood, urine, and PBMC samples and complete data for arsenic measures, %PTHMs, and potential confounders (*n* = 317). We also used all available PBMCs collected at baseline and week 12 from participants in the placebo group (*n* = 60) to examine if %PTHMs were altered after the use of arsenic-removal water filters: because of filter use, participants in the placebo group experienced a significant decrease in bAs concentrations from baseline to week 12 (Peters et al. 2015).

### General Characteristics

General characteristics of the study participants were determined at baseline by an in-person questionnaire. Body mass index (BMI) was calculated from the weight and height of each participant (kilograms/meters squared) measured at baseline.

### Total Blood Arsenic

As described previously (Peters et al. 2015), total bAs concentrations were measured using a Perkin-Elmer Elan DRC II ICP-MS equipped with an AS10+ autosampler. The intra- and interassay coefficients of variation (CVs) for bAs were 2.7% and 5.7%, respectively.

### Total Urinary Arsenic

We measured total urinary arsenic (uAs) by graphite furnace atomic absorption spectrophotometry, using an AAnalyst 600 graphite furnace system (Perkin Elmer), based on a method by Nixon et al. (1991). Intra- and inter-assay CVs for uAs were 3.1% and 5.4%, respectively. These values were adjusted for urinary creatinine (uCr) concentrations, which were measured using a method based on the Jaffe reaction (Slot 1965). The intra- and inter-assay CVs for uCr were 1.3% and 2.9%, respectively.

### Plasma Folate and B12

Plasma folate and B12 were measured using radio-protein binding assays (SimulTRAC-SNB, MP Biomedicals). The intra- and inter-assay CVs were 5% and 13%, respectively, for folate and 6% and 17%, respectively, for B12.

### Histone Isolation

We recently identified a cleavage product of histone H3 in human PBMCs that interferes with the measurement of PTHMs residing downstream of H3 cleavage sites (Howe and Gamble 2015); however, we note that the PTHMs in the current study are located upstream of cleavage sites and are therefore not affected by histone cleavage. Histones were isolated from PBMCs by acid extraction, as described previously (Chervona et al. 2012). Briefly, PBMCs were lysed for 10 min in radioimmunoprecipitation assay buffer supplemented with protease inhibitor E-64 to a concentration of 1 μM. The cell lysate was passed through a 21-gauge needle, and the pellet was collected by centrifugation, washed in histone washing buffer, collected again after centrifugation, and resuspended in 0.4 N H_2_SO_4_. After incubation at 4°C overnight, the supernatant was collected by centrifugation, mixed with acetone, and incubated overnight at –20°C. Pellets were collected by centrifugation, washed with acetone, dried, and resuspended in 4 M urea. Histone concentrations were determined by the Bradford Protein Assay according to the manufacturer’s instructions (Bio-Rad Laboratories, Hercules, CA). Samples were aliquoted and stored at –80°C.

### Determination of %H3K36me2, %H3K36me3, %H3K79me2

We measured %PTHMs using sandwich enzyme-linked immunosorbent assays (ELISAs), based on a previously described method (Chervona et al. 2012). Polystyrene 96-well microplates (Fisher Scientific) were coated with a capture antibody for total histone H3 (Abcam; 1:20,000) and incubated overnight at 4°C. The next day, plates were blocked with 3% milk diluted in phosphate-buffered saline with TWEEN-20 (PBST; 1× PBS, 0.05% TWEEN-20) for 2 hr and then washed with PBST. Histone samples from FACT participants were diluted with double-distilled water (ddH_2_O). Sample dilutions for each assay were as follows: H3K36me2, 1 ng/μL; H3K36me3, 1.5 ng/μL; H3K79me2, 2.0 ng/μL. A standard curve was generated with mixed histones from calf thymus (Sigma), and a pooled blood sample was included on each plate for calculating inter-assay CVs. FACT histone samples, calf histones, and the pooled blood sample were plated in duplicate. Plates were incubated on an orbital shaker at 20–22°C for 1.5 hr, and then the wells were washed with PBST. Detection antibodies were diluted in 1% milk in PBST to further prevent potential background signal, and 100 μL of the detection antibody solution was added to each well of the appropriate plate. Detection antibody dilutions were as follows: total H3 (Sigma), 1:40,000; H3K36me2 (Abcam), 1:2,000; H3K36me3 (Abcam), 1:2,000; H3K79me2 (Active Motif), 1:1,000. Plates were incubated for 1 hr at 20–22°C on an orbital shaker. Plates were washed with Tris-buffered saline with 0.1% TWEEN-20 (TBST), and 100 μL of secondary antibody [goat anti-rabbit immunoglobulin G–horseradish peroxidase (IgG-HRP) diluted 1:2,000 in TBS; Santa Cruz] was added to each well. Plates were incubated for 1 hr at 20–22°C without agitation. Subsequently, plates were washed with TBST followed by ddH_2_O, and 100 μL of 3,3´,5,5´-tetramethylbenzidine was added to each well. Plates were incubated in the dark for 10 min. The reaction was quenched with 2 N H_2_SO_4_, and the optical density was read at 450 nm using a SpectraMax 190 plate reader (Molecular Devices) with SoftMax Pro software (v.6.3). Total H3 and each of the three PTHMs were calculated relative to mixed calf histones based on a four-parameter logistic standard curve. The %PTHM was calculated by dividing the PTHM measure by the total H3 measure. Samples that were from the same individual but from different time points were run on the same plate. %H3K79me2 values were normalized to the pooled blood sample to reduce potential batch effects (Ramanakumar et al. 2010). The interassay CV for %H3K79me2 was calculated from a subset of samples (*n* = 16) measured on 2 separate days. The intra- and interassay CVs for each ELISA method were as follows: H3K36me2, 3.4% and 9.6%, respectively; H3K36me3, 4.9% and 11.9%, respectively; and H3K79me2, 7.1% and 7.0%, respectively. Because there were limited histone aliquots for the final assays, and because samples with poor reproducibility were excluded, the final sample sizes for %H3K36me2 (*n* = 311) and %H3K36me3 (*n* = 300) were smaller than the final sample size for %H3K79me2 (*n* = 315).

### Statistical Methods

Summary statistics were calculated for each variable [median (range) for continuous variables and percent for categorical variables] in all participants as well as separately by sex. Differences in continuous and categorical variables, both between men and women and between participants with and without %PTHM measures, were determined by Wilcoxon rank-sum and *χ* ^2^ tests, respectively. Transformations were applied to variables with skewed distributions to stabilize variances for parametric model assumptions and to reduce the influence of extreme values. A natural log transformation [ln(X)] was applied to each of the predictors, bAs and uAs, the latter of which was adjusted for uCr (uAs_Cr_); to the covariate BMI; and to two of the outcome variables, %H3K36me3, and %H3K79me2. An inverse transformation (1/Y) was applied to the third outcome variable, %H3K36me2.

A generalized linear model with an inverse-link function applied to the mean of (1/Y) was used to model the association between ln(bAs) or ln(uAs_Cr_) and the harmonic mean of %H3K36me2. Associations between the predictors, ln(bAs) and ln(uAs_Cr_), and the outcomes, ln(%H3K36me3) and ln(%H3K79me2), were examined using linear models. Arsenic regression coefficients (β) in models for %H3K36me2 indicated the change in the harmonic mean of %H3K36me2 for a unit increase in ln(bAs) or ln(uAs_Cr_), controlling for other variables in the model, and those for %H3K36me3 and %H3K79me2 indicated the change in the mean of the ln(%PTHM) for a unit increase in ln(bAs) or ln(uAs_Cr_), controlling for other variables in the model. Variables were considered potential confounders if they were correlated with arsenic exposure measures and the %PTHM in men or women and their addition to models changed arsenic exposure coefficients by > 10%. Therefore, we also present models adjusted for age, ln(BMI), education, and sex. To demonstrate the robustness of the associations between arsenic measures and %PTHMs, we also present analyses in the Supplemental Material with adjustments for additional variables: these include ln(uCr), ln(plasma folate), ln(plasma vitamin B12), and cigarette and betel nut use (ever vs. never). All variables except sex and education were included in models as continuous variables: the latter was dichotomized (education > 5 years vs. ≤ 5 years) because many participants had 0 years of education. Models were also run separately by sex, and differences by sex were determined using the Wald test, which compares regression coefficients between models (Clogg et al. 1995).

We also present Spearman correlations, which remain the same with or without applying the specified transformations to variables, showing the relationships between arsenic measures and %PTHMs to confirm that the directions of the associations are consistent with model-based results and to facilitate comparisons between %PTHMs.

Relationships between baseline and week 12 measures of each %PTHM were examined using Spearman correlations. The Wilcoxon signed-rank test was used to evaluate whether %PTHMs (untransformed) changed on average over a 12-week period; this test was performed in a subset of participants (*n* = 60) in the placebo group (*n* = 56 each for %H3K36me2 and %H3K79me2, *n* = 55 for %H3K36me3). We also examined the within-person changes in the %PTHMs separately by sex and tested for differences using the Wilcoxon rank-sum test.

A significance level of 0.05 was used for all statistical tests and regression models, which were performed using SAS (v.9.3, SAS Institute Inc.).

## Results

### General Characteristics, Arsenic Measures, and %PTHMs

General characteristics for the study participants are presented in Table 1. Participants were between 24 and 54 years old. Approximately 22.4% of the study participants had > 5 years of education. Blood arsenic concentrations ranged from 1.0 to 80.2 μg/L. Concentrations of uAs_Cr_ ranged from 35 to 2,200 μg/g uCr. Compared with women, male study participants were older, had lower BMIs, and had higher uCr and bAs concentrations and lower uAs_Cr_ concentrations. Men were also more likely to have low plasma folate concentrations and to be cigarette smokers. Individuals in the placebo group with %PTHM measures (see Table S1) were very similar to the overall study population (Table 1). The only variables that differed were uCr concentrations and, consequently, uAs concentrations, which were both lower in the placebo group participants. However, uAs_Cr_ concentrations were similar between groups.

**Table 1:** Baseline characteristics*^a^* of FACT participants with at least one %PTHM measure and complete information for variables included in regression models.

Characteristic	Whole sample (*n* = 317)	Men (*n* = 161)	Women (*n* = 156)	*p*-Value^*b*^
Age (years)	39 (24–54)	42 (25–54)	37 (24–54)	< 0.01
BMI (kg/m^2^)	19.3 (13.9–31.6)	18.7 (15.4–27.9)	20.0 (13.9–31.6)	< 0.01
uCr (μg/L)	48 (6–252)	53 (6–252)	45 (6–233)	0.03
bAs (μg/L)	8.8 (1.0–80.2)	9.6 (2.5–52.0)	7.9 (1.0–80.2)	0.05
uAs (μg/L)	121 (11–1,770)	123 (11–1,770)	121 (11–1,320)	0.67
uAs_Cr_ (μg/g uCr)	257 (35–2,200)	242 (65–1,480)	287 (35–2,200)	0.03
%H3K36me2^*c*^	1.45 (0.68–6.87)	1.45 (0.68–4.00)	1.43 (1.00–6.87)	0.47
%H3K36me3^*d*^	1.61 (0.48–6.44)	1.57 (0.48–4.09)	1.62 (0.52–6.44)	0.18
%H3K79me2^*e*^	1.26 (0.29–9.46)	1.26 (0.29–9.46)	1.25 (0.29–9.41)	0.69
Folate < 9 nmol/L	74 (23.3)	46 (28.6)	28 (18.0)	0.03
B12 < 151 pmol/L	77 (24.3)	39 (24.2)	38 (24.4)	0.98
Ever smoker	93 (29.3)	91 (56.5)	2 (1.3)	< 0.01
Ever betel	87 (27.4)	48 (29.8)	39 (25.0)	0.34
Education > 5 years	77 (22.4)	33 (20.5)	38 (24.4)	0.41
Abbreviations: bAs, blood arsenic; BMI, body mass index; FACT, Folic Acid and Creatine Trial; H3K36me2, dimethylation of lysine 36 of histone H3; H3K36me3, trimethylation of lysine 36 of histone H3; H3K79me2, dimethylation of lysine 79 of histone H3; PTHM, post-translational histone modification; uAs, urinary arsenic; uAs_Cr_, urinary arsenic adjusted for urinary creatinine; uCr, urinary creatinine. ^***a***^Values are median (range) or *n* (%) for continuous and categorical variables, respectively. ^***b***^Wilcoxon rank-sum or χ^2^ test for difference between men and women in continuous and categorical variables, respectively. ^***c***^*n *= 311 (men: 158, women: 153). ^***d***^*n *= 300 (men: 153, women: 147). ^***e***^*n *= 315 (men: 161, women: 154).				

FACT participants with %PTHM measures were generally comparable to FACT participants without %PTHM measures (see Table S2), although participants with %PTHM measures were slightly older, were more likely to have low folate, and had higher uCr and uAs concentrations (before adjustment for uCr).

### Associations Between Arsenic Exposure and %PTHMs

In the whole sample, neither ln(bAs)nor ln(uAs_Cr_) was significantly associated with any of the %PTHMs (Table 2). However, ln(uAs_Cr_) was positively associated with %H3K36me2 in men both before [β = 0.13; 95% confidence interval (CI): 0.02, 0.24; *p* = 0.02] and after (β = 0.12; 95% CI: 0.01, 0.23; *p* = 0.03) adjusting for age, education, and ln(BMI). The association between ln(uAs_Cr_) and %H3K36me2 was in the opposite direction for women (covariate-adjusted β = –0.05; 95% CI: –0.12, 0.02; *p* = 0.19) and differed significantly from the corresponding estimate in men (*p* = 0.01) (Table 2). Although not statistically significant, associations between ln(bAs) and %H3K36me2 were similar to those for ln(uAs_Cr_), with estimates that were positive in men and negative in women (*p* for difference between men and women = 0.08 for covariate-adjusted models). The patterns of associations according to sex were similar for ln(%H3K36me3). Because coefficients in models for %H3K36me2 represent changes in the harmonic mean of %H3K36me2, whereas coefficients in models for %H3K36me3 represent changes in the mean of ln(%H3K36me3), the magnitudes of the associations cannot be directly compared. However, the findings were consistent when examined by Spearman correlation, which does not require that variables be transformed and thus allows for more direct comparisons to be made between %PTHMs (see Table S3). Although associations between ln-transformed arsenic measures and ln(%H3K36me3) were not significant in either men or women, differences by sex were significant or suggestive (Table 2). Furthermore, ln-transformed arsenic measures were not associated with ln(%H3K79me2) in men or in women, and differences by sex were not significant (Table 2). Associations between arsenic measures and %PTHMs were highly similar after additionally adjusting for ln(uCr), ln(plasma folate), ln(plasma B12), cigarette smoking status, and betel nut use (see Table S4).

**Table 2:** Estimated regression coefficients*^a^* and 95% confidence intervals for associations between baseline measures of arsenic exposure and %PTHMs in FACT participants.

%PTHM/arsenic exposure	Whole sample	Men	Women	*p*-Value^*b*^
%H3K36me2^*c*^
bAs	0.02 (–0.05, 0.09)	0.12 (–0.00, 0.24)	–0.04 (–0.12, 0.04)	0.04
Adjusted bAs^*d*^	0.02 (–0.05, 0.09)	0.10 (–0.02, 0.22)	–0.03 (–0.11, 0.05)	0.08
uAs_Cr_	0.01 (–0.05, 0.08)	0.13 (0.02, 0.24)*	–0.05 (–0.12, 0.02)	< 0.01
Adjusted uAs_Cr_^*d*^	0.02 (–0.05, 0.08)	0.12 (0.01, 0.23)*	–0.05 (–0.12, 0.02)	0.01
%H3K36me3^*e*^
bAs	–0.03 (–0.10, 0.04)	0.06 (–0.04, 0.16)	–0.08 (–0.17, 0.01)	0.04
Adjusted bAs^*d*^	–0.02 (–0.09, 0.05)	0.05 (–0.04, 0.15)	–0.07 (–0.16, 0.02)	0.07
uAs_Cr_	–0.01 (–0.07, 0.05)	0.07 (–0.02, 0.16)	–0.06 (–0.14, 0.02)	0.04
Adjusted uAs_Cr_^*d*^	0.00 (–0.06, 0.06)	0.07 (–0.02, 0.16)	–0.05 (–0.14, 0.03)	0.05
%H3K79me2^*f*^
bAs	0.04 (–0.05, 0.12)	0.04 (–0.09, 0.17)	0.03 (–0.08, 0.14)	0.91
Adjusted bAs^*d*^	0.03 (–0.05, 0.12)	0.04 (–0.09, 0.17)	0.04 (–0.08, 0.15)	0.95
uAs_Cr_	0.02 (–0.06, 0.09)	0.05 (–0.07, 0.17)	0.00 (–0.10, 0.10)	0.53
Adjusted uAs_Cr_^*d*^	0.01 (–0.06, 0.09)	0.04 (–0.08, 0.17)	0.01 (–0.10, 0.11)	0.65
Abbreviations: bAs, blood arsenic; FACT, Folic Acid and Creatine Trial; H3K36me2, dimethylation of lysine 36 of histone H3; H3K36me3, trimethylation of lysine 36 of histone H3; H3K79me2, dimethylation of lysine 79 of histone H3; PTHM, post-translational histone modification; uAs_Cr_, urinary arsenic adjusted for urinary creatinine. ^***a***^Estimated regression coefficients and 95% confidence intervals [β (CI)] from generalized linear models. Associations were examined between ln(bAs) or ln(uAs_Cr_) in relation to each of the three %PTHMs. Coefficients from %H3K36me2 models indicate the change in the harmonic mean of %H3K36me2 for a unit increase in the natural log–transformed arsenic measure, controlling for other covariates. Coefficients from %H3K36me3 and %H3K79me2 models indicate the change in the mean of the ln(%PTHM) for a unit increase in the natural log–transformed arsenic measure, controlling for other covariates. ^***b***^Wald test for sex difference. ^***c***^Whole sample *n *= 311, men *n *= 158, women *n *= 153. ^***d***^Adjusted for age, education (dichotomized at 5 years), and ln(BMI). Whole-sample analyses were also adjusted for sex. ^***e***^Whole sample *n *= 300, men *n *= 153, women *n *= 147. ^***f***^Whole sample *n *= 315, men *n *= 161, women *n *= 154. *p < 0.05.				

### Stability of %PTHMs after Reductions in Arsenic Exposure

The use of arsenic-removal water filters for 12 weeks was associated with significant reductions in bAs (see Peters et al. 2015) and uAs_Cr_ (*p* < 0.01, Wilcoxon signed-rank test) in the placebo group. Summary statistics for within-person changes in %H3K36me2, %H3K36me3, and %H3K79me2 from baseline to week 12 for participants in the placebo group are presented in Table 3. Although baseline values were significantly correlated with values measured at week 12 for each of the three %PTHMs analyzed (*p*-values from Spearman correlations < 0.01), the median change in %H3K36me2 from baseline to week 12 was negative (–0.15). Thus, on average, %H3K36me2 declined over time (*p* < 0.01), although the interquartile range (IQR) for the within-person change (–0.43, 0.11) indicated that this mark did increase over time in ≥ 25% of the participants. In sex-stratified analyses, %H3K36me2 was found to decrease on average among both men and women. However, the decline was only statistically significant among women (*p* < 0.01). There was no significant change in %H3K36me3 during the 12 week period, but there was a suggestive decrease in %H3K79me2 (median within-person change: –0.05; IQR: –0.24, 0.04, *p* = 0.07). The within-person changes in %PTHMs did not differ significantly by sex (Table 3).

**Table 3:** Within-person changes in %PTHMs from baseline to week 12 for FACT participants in the placebo group.

%PTHM	Whole sample			Men			Women			*p*-Value^*a*^
	*n*	Median (IQR)	*p*-Value^*b*^	*n*	Median (IQR)	*p*-Value^*b*^	*n*	Median (IQR)	*p*-Value^*b*^	
%H3K36me2	56	–0.15 (–0.43, 0.11)	< 0.01	27	–0.07 (–0.44, 0.16)	0.14	29	–0.17 (–0.37, 0.04)	< 0.01	0.62
%H3K36me3	55	0.02 (–0.23, 0.30)	0.61	28	0.05 (–0.23, 0.47)	0.35	27	0.02 (–0.22, 0.12)	0.93	0.35
%H3K79me2	56	–0.05 (–0.24, 0.04)	0.07	29	–0.03 (–0.28, 0.09)	0.36	27	–0.05 (–0.18, 0.04)	0.10	0.88
Abbreviations: FACT, Folic Acid and Creatine Trial; H3K36me2, dimethylation of lysine 36 of histone H3; H3K36me3, trimethylation of lysine 36 of histone H3; H3K79me2, dimethylation of lysine 79 of histone H3; IQR, interquartile range; PTHM, post-translational histone modification. ^***a***^Wilcoxon rank-sum test for difference between men and women in the within-person change for each %PTHM. ^***b***^Wilcoxon signed-rank test for within-person change in %PTHM.										

## Discussion

In an adult population in Bangladesh, we examined associations between arsenic exposure and three %PTHMs (%H3K36me2, %H3K36me3, and %H3K79me2), which were selected because they are dysregulated in several types of cancer (Bernt et al. 2011; Duns et al. 2010; Fontebasso et al. 2013; Tamagawa et al. 2013; Zhang L et al. 2014) and are altered by arsenic *in vitro* (Zhou et al. 2008) or because they regulate 5-hmC (Huang et al. 2013; Williams et al. 2011). Percent 5-hmC has been shown to be altered by arsenic in male rats (Zhang J et al. 2014) and in humans in a sex-dependent manner (Niedzwiecki et al. 2015). We observed that arsenic was associated with higher levels of %H3K36me2, but only in men. Interestingly, the use of arsenic-removal water filters, which was associated with significant reductions in both bAs (Peters et al. 2015) and uAsCr, was also associated with significant reductions in %H3K36me2 in the whole sample. However, in sex-stratified analyses, although we observed that %H3K36me2 declined in both men and women, the decline only achieved statistical significance among women. Given that ethical considerations did not allow for a comparison group that did not receive arsenic-removal water filters, we cannot rule out the possibility that the decline in %H3K36me2 may have been caused by extrinsic factors. Additional studies will be needed to confirm the changes we observed in %H3K36me2 in association with arsenic removal. In cross-sectional analyses, arsenic exposure was not associated with %H3K36me3. Additionally, %H3K36me3 did not change over time despite reductions in arsenic exposure. Thus, arsenic did not appear to alter %H3K36me3 in histones derived from PBMCs. Although in cross-sectional analyses arsenic exposure was not associated with %H3K79me2, which has been shown to regulate the expression of Tet1 (Huang et al. 2013; Williams et al. 2011), we did observe a suggestive (*p* = 0.07) decline in %H3K79me2 over time after individuals received arsenic-removal water filters; this observation needs to be confirmed in a larger sample.

Although neither %H3K36me3 nor %H3K79me2 changed significantly over the 12-week period, we cannot make definitive conclusions about the stability of these marks because all participants received arsenic-removal water filters at baseline and were thus subject to an intervention; furthermore, these marks did vary over time in some participants. Little is known about the stability of %PTHMs in human PBMCs. One study by Zhang et al. examined the stability of PTHMs during adipogenesis, and the authors observed that although gene-specific levels of PTHMs were highly dynamic, global levels of PTHMs were remarkably stable (Zhang et al. 2012). However, because Zhang et al. used murine adipocyte cell lines, it is unclear if these findings are relevant to %PTHM stability in human PBMCs. One previous epidemiological study measured %PTHMs in PBMCs collected at three 1-week intervals from 15 nickel refinery workers and from 15 individuals who had not been exposed occupationally to nickel, and the authors observed that the interindividual variances in %PTHMs were much higher than the intraindividual variances, suggesting that %PTHMs are relatively stable over time in human PBMCs (Arita et al. 2012). However, because this evaluation was made over a short duration and in a small number of participants, this area requires additional investigation.

Our study has several potential limitations. First, given the cross-sectional nature of some of the analyses, we need to consider the possibility of reverse causality. Because several previous experimental studies have shown that arsenic influences %PTHMs, it is unlikely that reverse causality would explain our findings. However, it is possible that %PTHMs influence the expression of genes involved in arsenic metabolism, such as arsenic (+3 oxidation state) methyltransferase, which could thereby influence the excretion of arsenic, thus altering bAs and uAs_Cr_ concentrations. Although residual confounding is another important consideration, the associations between arsenic measures and %PTHMs were quite robust even after adjusting for additional covariates. Another important consideration for our study is the fact that %PTHMs were measured in human PBMCs, which comprise a mixed population of cell types. However, global DNA methylation levels, which are closely related to %PTHMs, have been shown to be very similar between blood cell types (reviewed in Smith and Meissner 2013). Additionally, a cross-sectional study of 63 male steel workers that examined associations between inhalation exposure to occupational toxicants, including arsenic, and %PTHMs in total WBCs evaluated the influence of cell-type distribution on these associations, and the authors observed that although adjusting for the proportion of granulocytes influenced their results, adjusting for other cell types did not have a major impact on the findings (Cantone et al. 2011). Because we measured %PTHMs in PBMCs, which do not include granulocytes, potential shifts in the proportion of granulocytes could not explain the associations we observed between arsenic and %PTHMs. However, we cannot rule out the possibility that alterations in the proportion of monocytes, natural killer cells, T cells, B cells, or their subpopulations may have affected our findings, and this is an area that merits additional investigation. Finally, an important limitation of our study is that the sample sizes for prospective analyses were small. Therefore, we may have had insufficient statistical power to formally examine sex differences in the influence of arsenic removal on %PTHMs.

Despite some of the limitations of this study, our findings support those of a previous experimental study performed in A549 cells that examined the effects of arsenite (2.5 and 5 μM) on %H3K36me2 and %H3K36me3 (Zhou et al. 2008); in that study, the authors observed that arsenic decreased %H3K36me2 and increased %H3K36me3. Although we observed a positive association between uAs_Cr_ and %H3K36me2 in men and did not observe an association between uAs_Cr_ and %H3K36me3, we studied a population that had been exposed to arsenic-contaminated drinking water for years to decades, whereas Zhou et al. measured %PTHMs in a cell line that had been exposed to arsenic for a 24-hr period. Furthermore, our study participants were healthy individuals, whereas A549 cells are alveolar basal epithelial cells derived from a male human lung tumor; arsenic may have distinct effects in different tissues and may also have different effects in normal versus cancerous cells. Additionally, *in vitro* studies are limited in that they cannot account for the numerous systemic differences associated with sex *in vivo*. Nevertheless, it is quite interesting that arsenic appears to target H3K36me2 in such diverse models.

Although the consequences of arsenic-induced increases in %H3K36me2 are unknown at the present time, H3K36me2 has been implicated in oncogenic programming (Kuo et al. 2011). Furthermore, some of the enzymes responsible for regulating this mark, such as methyltransferase NSD2, are overexpressed in multiple cancers, including those caused by arsenic, such as bladder, lung, and skin cancers (Hudlebusch et al. 2011). A global increase in H3K36me2 leads to widespread increases in this mark across the genome, thereby altering its typical distribution (Popovic et al. 2014), which may have profound effects on both gene expression and genomic stability. For example, a global increase in H3K36me2 is associated with increased levels of H3K36me2 within gene bodies, which in turn is associated with increased expression of genes involved in oncogenic programming (Kuo et al. 2011).

Similar to our findings, several studies have observed sex-specific effects of arsenic on other epigenetic marks, such as DNA methylation (Broberg et al. 2014; Nohara et al. 2011; Pilsner et al. 2012), including our previous finding that arsenic exposure is associated with increased %5-mC and %5-hmC in men but not in women (Niedzwiecki et al. 2015). Sex-specific effects of other environmental contaminants, such as cadmium and lead, on DNA methylation have also been observed (Faulk et al. 2013; Kippler et al. 2013). Because PTHMs can direct DNA methylation patterns (Cedar and Bergman 2009), PTHMs may mediate the effects of environmental contaminants, such as arsenic, on DNA methylation marks. In addition to the sex-specific findings for uAs_Cr_ and %H3K36me2 reported herein, our group previously observed sex-specific correlations between uAs_Cr_ and several other %PTHMs (Chervona et al. 2012). Additionally, Tyler et al. recently demonstrated that arsenic alters %PTHMs in a sex-dependent manner in mouse brain (Tyler et al. 2015). Although the mechanisms are not fully understood, arsenic also altered the expression of corresponding histone-modifying enzymes, including *Mll* and *Kdm5b*, in a sex-dependent manner (Tyler et al. 2015). Many histone-modifying enzymes have also been shown to interact with the androgen receptor (Heemers and Tindall 2007), and some histone demethylases are dosage-sensitive regulators that are coded for by genes that reside on the Y chromosome and are highly conserved across mammalian species and broadly expressed across tissues and cell types (Bellott et al. 2014). Thus, both hormonal influences and genetic differences may contribute to the sex-specific effects of arsenic on %PTHMs.

For many arsenic-induced health outcomes, susceptibility differs by sex (NRC 2013). For example, men are more susceptible to developing arsenic-induced skin lesions (Ahsan et al. 2006b; Watanabe et al. 2001) and cancers of the skin, liver, and bladder (Chen and Wang 1990; Chen et al. 2003; Leonardi et al. 2012). In contrast, several studies have reported that early-life exposure to arsenic is associated with impaired intellectual function and other developmental outcomes among female, but not male, children (Gardner et al. 2013; Hamadani et al. 2011; Saha et al. 2012). Additionally, in the United States, the arsenic-associated risk for CVD was found to be higher among women (Moon et al. 2013). Animal studies have also revealed sex-specific effects of arsenic for many outcomes. For example, female mice are more susceptible to arsenic-induced changes in locomotor activity (Bardullas et al. 2009) and are more likely to develop lung tumors as a result of prenatal exposure to arsenic, whereas males are more likely to develop liver and adrenal tumors in response to such exposures (Waalkes et al. 2003).

Although some of the sex-specific effects of arsenic observed in human populations may be explained by sex differences in coexposures (e.g., cigarette smoking, UV exposure, and nutritional deficiencies), the dramatic sex differences observed in well-controlled animal studies of arsenic toxicity suggest that coexposures are not solely responsible for these differences. One consideration is that women have a higher capacity to fully methylate inorganic arsenic to dimethyl arsenic species, which facilitates arsenic elimination in urine (Hopenhayn-Rich et al. 1996; Hsueh et al. 2003; Lindberg et al. 2007), which should generally reduce arsenic toxicity for women. However, some arsenic-related health outcomes preferentially afflict women; thus, there are likely other contributing factors. Epigenetic dysregulation has been implicated in the development of arsenic-induced health outcomes, including skin lesions and cancers of the skin and bladder (Chanda et al. 2006; Pilsner et al. 2009; Smeester et al. 2011; Wilhelm et al. 2010). Thus, epigenetic dysregulation may be one important mechanism contributing to the sex differences observed for multiple arsenic-related health outcomes. Previous studies examining the sex-specific effects of arsenic on epigenetics have focused on DNA methylation. However, the results of this study and those of our previous study (Chervona et al. 2012) suggest that arsenic also induces sex-specific alterations in %PTHMs.

## Conclusions

Our findings have two major implications that warrant further investigation. First, arsenic exposure was associated with %H3K36me2 in a sex-dependent manner in our study population of adults in Bangladesh. Although it is tempting to speculate that these findings may explain some of the observed sex differences in susceptibility to arsenic-induced diseases, the impact of %H3K36me2 and other %PTHMs on health outcomes will require further study. Second, the arsenic-associated increase in %H3K36me2 observed in men decreased, albeit non-significantly, after the use of arsenic-removal water filters. However, because we did not have a comparison group that did not receive water filters, and because %H3K36me2 decreased significantly in women, future studies will be needed to evaluate the effects of arsenic removal on %H3K36me2 and to investigate whether downstream effects of alterations in %PTHMs, such as changes in DNA methylation patterns, persist over time.

## Supplemental Material

(304 KB) PDF
