# Supplementary material for: Associations between Blood and Urine Arsenic Concentrations and Global Levels of Post-Translational Histone Modifications in Bangladeshi Men and Women
Source: Environ Health Perspect. 2016 Mar 11;124(8):1234–40. doi: 10.1289/ehp.1510412 (PMC4977054; doi:10.1289/ehp.1510412)
Supplement: (304 KB) PDF [file ehp.1510412.s001.acco.pdf]

Environ Health Perspect
DOI: 10.1289/ehp.1510412

Note to readers with disabilities: *EHP* strives to ensure that all journal content is accessible to all readers. However, some figures and Supplemental Material published in *EHP* articles may not conform to 508 standards due to the complexity of the information being presented. If you need assistance accessing journal content, please contact ehp508@niehs.nih.gov. Our staff will work with you to assess and meet your accessibility needs within 3 working days.

Supplemental Material

Associations between Blood and Urine Arsenic Concentrations and Global Levels of Post-Translational Histone Modifications in Bangladeshi Men and Women

Caitlin G. Howe, Xinhua Liu, Megan N. Hall, Vesna Slavkovich, Vesna Ilievski, Faruque Parvez, Abu B. Siddique, Hasan Shahriar, Mohammad N. Uddin, Tariqul Islam, Joseph H. Graziano, Max Costa, and Mary V. Gamble

Table of Contents

Table S1. Baseline characteristics of FACT participants in the placebo group with %PTHM measures

Table S2. Baseline characteristics of FACT participants with vs. without %PTHM measures and with complete information for variables included in regression models

Table S3. Spearman correlation coefficients for baseline measures of arsenic exposure and %PTHMs in FACT participants

Table S4. Estimated regression coefficients and 95% confidence intervals for associations between baseline measures of arsenic exposure and %PTHMs, after adjusting for additional covariates, in FACT participants

Figure S1. Folic Acid and Creatine Trial (FACT) Study Design and Sampling for Current Study. **(A)** FACT study design. A total of 622 participants were randomized to five treatment arms. 12 participants were dropped during the course of the study. The final sample size for each

treatment arm is shown. Approximately half of the participants in each folic acid (FA) treatment arm were switched to placebo at week 12. All participants received arsenic-removal water filters at baseline to be used for the duration of the 24 week study period and thereafter. **(B)** The first set of analyses for the current study used peripheral blood mononuclear cell (PBMC) samples collected at baseline from a total of 317 FACT participants. We show here the distribution of the 317 participants by treatment arm. By design, the majority of participants in the current study were from the placebo and the 400 μg FA/day treatment arms. To ensure sufficient statistical power for regression analyses, an additional 123 PBMC samples were selected from participants in other treatment arms who had measures for all covariates of interest. All cross-sectional analyses for the current study used PBMC samples that were collected from these 317 participants at baseline, prior to the provision of arsenic-removal water filters and nutritional supplements or a placebo **(C)** The second set of analyses for the current study used all available samples from the placebo group (n = 60). These samples were collected at baseline and at week 12. Participants in the placebo group did not receive any nutritional interventions during the study period but, like all other FACT participants, received arsenic-removal water filters at baseline to be used during the study period and thereafter.

Table S1. Baseline characteristics[a] of FACT participants in the placebo group with %PTHM measures

**Characteristic**	**Whole Sample (n = 60)**	**Men (n = 31)**	**Women (n = 29)**	***p*[b]**
Age (years)	38 (25-53)	39 (25-53)	35 (27-52)	0.37
BMI (kg/m^2)[c]	19.5 (15.4-31.6)	18.7 (15.4-24.0)	21.3 (16.6-31.6)	<0.01
uCr (μg/L)	40 (6-121)	33 (6-121)	45 (8-102)	0.26
bAs (μg/L)	8.7 (1.0-34.7)	9.7 (3.9-17.8)	8.0 (1.0-34.7)	0.24
uAs (μg/L)	88 (11-796)	71 (11-305)	116 (22-796)	0.16
uAs_{Cr} (μg/g uCr)	297 (35-1250)	245 (91-598)	297 (35-1250)	0.33
%H3K36me2[d]	1.56 (0.68-3.86)	1.60 (0.68-3.86)	1.52 (1.03-2.33)	0.20
%H3K36me3[e]	1.67 (0.80-4.16)	1.73 (0.80-3.94)	1.66 (0.85-4.16)	0.87
%H3K79me2[f]	1.22 (0.66-3.46)	1.16 (0.29-2.78)	1.22 (0.66-3.46)	0.75
Folate < 9 nmol/L	11 (18.3)	7 (22.6)	4 (13.8)	0.51[g]
B12 < 151 pmol/L	16 (26.7)	7 (22.6)	9 (31.0)	0.65
Ever Smoker	19 (31.7)	19 (61.3)	0 (0.0)	<0.01[g]
Ever Betel	16 (26.7)	7 (22.6)	9 (31.0)	0.65
Education > 5 y	10 (16.7)	3 (9.7)	7 (24.1)	0.17[g]

Abbreviations: bAs, blood arsenic; BMI, body mass index; FACT, Folic Acid and Creatine Trial; H3K36me2, di-methylation of lysine 36 of histone H3; H3K36me3, tri-methylation of lysine 36 of histone H3; H3K79me2, di-methylation of lysine 79 of histone H3; PTHM, post-translational histone modification; uAs, urinary arsenic; uAs_{Cr}, urinary arsenic adjusted for urinary creatinine; uCr, urinary creatinine

[a]Values are median (range) or n (%) for continuous and categorical variables, respectively

[b]Wilcoxon rank-sum test or Chi-square test for difference between men and women in continuous and categorical variables, respectively

[c]n = 28 for women

[d]n = 58 for whole sample, n = 29 for men

[e]n = 57 for whole sample, n = 29 for men, n = 28 for women

[f]n = 60 for whole sample, n = 31 for men, n = 29 for women

[g]Fisher's exact test

Table S2. Baseline characteristics[a] of FACT participants with vs. without %PTHM measures and with complete information for variables included in regression models

Characteristic	Participants with %PTHM Measures (n = 317)	Participants without %PTHM Measures (n = 293)	p^b
Age (years)	39 (24-54)	37 (24-55)	0.03
BMI (kg/m^2)	19.3 (13.9-31.6)	19.6 (14.3-27.6)	0.27
uCr (µg/L)	48 (6-252)	41 (6-303)	<0.01
bAs (µg/L)	8.8 (1.0-80.2)	8.8 (1.8-35.0)	0.43
uAs (µg/L)	121 (11-1770)	109 (7-769)	0.05
uAs_{Cr} (µg/g uCr)	257 (35-2200)	275 (46-1100)	0.35
Men	161 (50.8)	146 (49.8)	0.81
Folate < 9 nmol/L	74 (23.3)	46 (15.7)	0.02
B12 < 151 pmol/L	77 (24.3)	67 (22.9)	0.68
Ever Smoker	93 (29.3)	73 (24.9)	0.22
Ever Betel	87 (27.4)	63 (21.5)	0.09
Education > 5 y	71 (22.4)	83 (28.3)	0.09

Abbreviations: bAs, blood arsenic; BMI, body mass index; FACT, Folic Acid and Creatine Trial; PTHM, post-translational histone modification; uAs, urinary arsenic; uAs_{Cr}, urinary arsenic adjusted for urinary creatinine; uCr, urinary creatinine

[a]Values are median (range) or n (%) for continuous and categorical variables, respectively

[b]Wilcoxon rank-sum or Chi-square test for difference between those with vs. without %PTHM measures for continuous and categorical variables, respectively

Table S3. Spearman correlation coefficients[a] for baseline measures of arsenic exposure and %PTHMs in FACT participants

**%PTHM**	**Whole Sample**		**Men**		**Women**	
	bAs	uAs_{Cr}	bAs	uAs_{Cr}	bAs	uAs_{Cr}
Unadjusted						
%H3K36me2[b]	0.04 (0.44)	0.02 (0.68)	0.18 (0.02)	0.21 (<0.01)	-0.11 (0.18)	-0.13 (0.10)
%H3K36me3[c]	-0.01 (0.85)	0.01 (0.81)	0.09 (0.26)	0.11 (0.18)	-0.10 (0.22)	-0.09 (0.28)
%H3K79me2[d]	0.02 (0.68)	0.00 (0.97)	0.02 (0.78)	0.04 (0.61)	0.03 (0.74)	-0.03 (0.70)
Adjusted[e]						
%H3K36me2[b]	0.04 (0.50)	0.03 (0.59)	0.17 (0.04)	0.20 (0.01)	-0.11 (0.17)	-0.15 (0.06)
%H3K36me3[c]	0.00 (0.98)	0.01 (0.85)	0.08 (0.33)	0.11 (0.16)	-0.09 (0.26)	-0.09 (0.30)
%H3K79me2[d]	0.01 (0.88)	-0.01 (0.85)	0.02 (0.76)	0.03 (0.69)	0.03 (0.74)	-0.02 (0.77)

Abbreviations: bAs, blood arsenic; FACT, Folic Acid and Creatine Trial; H3K36me2, di-methylation of lysine 36 of histone H3; H3K36me3, tri-methylation of lysine 36 of histone H3; H3K79me2, di-methylation of lysine 79 of histone H3; PTHM, post-translational histone modification; uAs^{Cr}, urinary arsenic adjusted for urinary creatinine

[a]Values presented are Spearman correlation coefficients, with respective *p*-values indicated in parentheses

[b]n = 311 for whole sample, n = 158 for men, n = 153 for women

[c]n = 300 for whole sample, n = 153 for men, n = 147 for women

[d]n = 315 for whole sample, n = 161 for men, n = 154 for women

[e]Partial Spearman correlation coefficients, adjusted for age, education, body mass index. Whole sample analyses were also adjusted for sex.

Table S4. Estimated regression coefficients[a] and 95% confidence intervals for associations between baseline measures of arsenic exposure and %PTHMs, after adjusting for additional covariates, in FACT participants

**%PTHM**	**Arsenic Exposure**	**Whole Sample**	**Men**	**Women**	***p*[b]**
%H3K36me2[c]	bAs	0.02 (-0.05, 0.09)	0.10 (-0.02, 0.22)	-0.04 (-0.12, 0.04)	0.06
	uAs_{Cr}	0.01 (-0.05, 0.08)	0.12 (+0.00, 0.23)*	-0.06 (-0.14, 0.02)	0.01
%H3K36me3[d]	bAs	0.00 (-0.07. 0.06)	0.06 (-0.05, 0.16)	-0.05 (-0.15, 0.04)	0.12
	uAs_{Cr}	-0.01 (-0.08, 0.05)	0.07 (-0.03, 0.17)	-0.04 (-0.13, 0.05)	0.10
%H3K79me2[e]	bAs	0.04 (0.05, 0.12)	0.06 (-0.07, 0.19)	0.03 (-0.08, 0.14)	0.75
	uAs_{Cr}	0.02 (-0.06, 0.10)	0.06 (-0.07, 0.19)	0.00 (-0.10, 0.11)	0.49

Abbreviations: bAs, blood arsenic; FACT, Folic Acid and Creatine Trial; H3K36me2, di-methylation of lysine 36 of histone H3; H3K36me3, tri-methylation of lysine 36 of histone H3; H3K79me2, di-methylation of lysine 79 of histone H3; PTHM, post-translational histone modification; uAs_{Cr}, urinary arsenic adjusted for urinary creatinine

[a]Estimated regression coefficients and 95% confidence intervals (β (CI)) from generalized linear models. Associations were examined between ln-bAs or ln-uAs_{Cr} in relation to each of the three %PTHMs. Coefficients from %H3K36me2 models indicate the change in the harmonic mean of %H3K36me2 for a unit increase in the ln-transformed arsenic measure, controlling for other covariates. Coefficients from %H3K36me3 and %H3K79me2 models indicate the change in the mean of the ln-%PTHM for a unit increase in the ln-transformed arsenic measure, controlling for other covariates. Models were adjusted for age, education (dichotomized at 5 years), ln-BMI, ln-uCr, ln-plasma folate, ln-plasma vitamin B12, cigarette smoking status (ever vs. never), and betel nut chewing status (ever vs. never). Whole sample analyses were also adjusted for sex.

[b]Wald test for sex difference

[c]Whole sample n = 311, Males n = 158, Females n = 153.

[d]Whole sample n = 300, Males n = 153, Females n = 147.

[e]Whole sample n = 315, Males n = 161, Females n = 154.

*$p < 0.05$

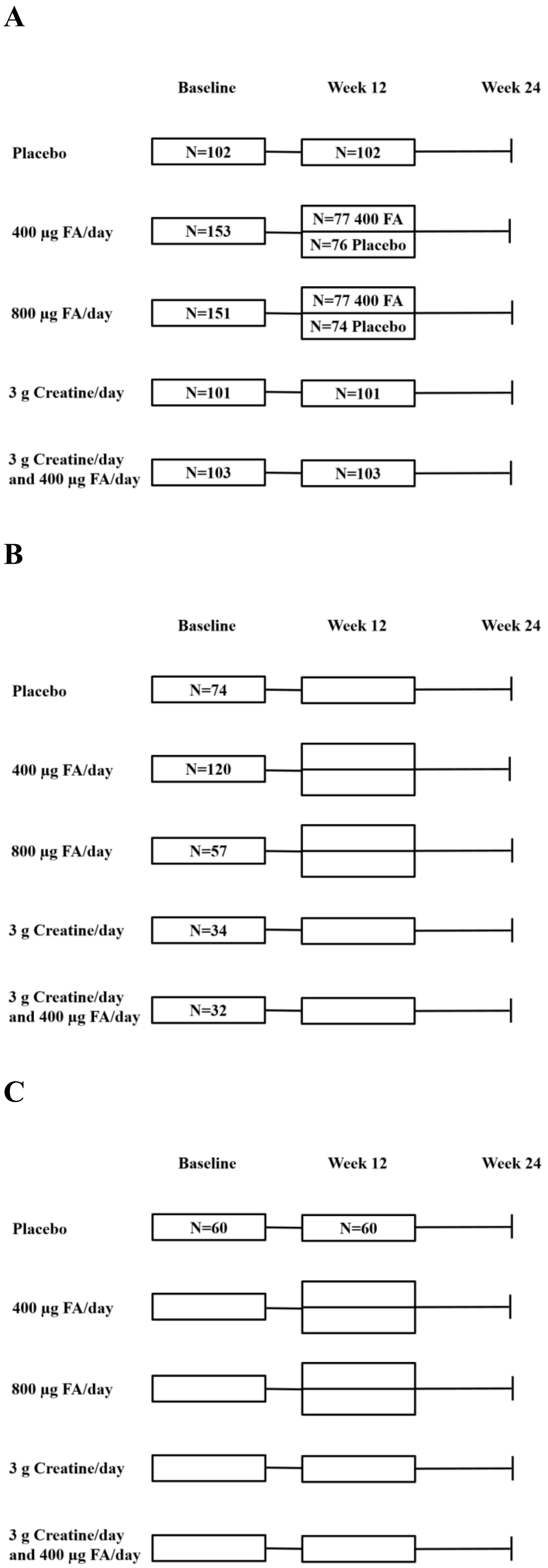
A
Baseline
Week 12
Week 24
Placebo
N=102
N=102
400 µg FA/day
N=153
N=77 400 FA
N=76 Placebo
800 µg FA/day
N=151
N=77 400 FA
N=74 Placebo
3 g Creatine/day
N=101
N=101
3 g Creatine/day and 400 µg FA/day
N=103
N=103
B
Baseline
Week 12
Week 24
Placebo
N=74
400 µg FA/day
N=120
800 µg FA/day
N=57
3 g Creatine/day
N=34
3 g Creatine/day and 400 µg FA/day
N=32
C
Baseline
Week 12
Week 24
Placebo
N=60
N=60
400 µg FA/day
800 µg FA/day
3 g Creatine/day
3 g Creatine/day and 400 µg FA/day

Figure S1. Folic Acid and Creatine Trial (FACT) Study Design and Sampling for Current Study. **(A)** FACT study design. A total of 622 participants were randomized to five treatment arms. 12 participants were dropped during the course of the study. The final sample size for each treatment arm is shown. Approximately half of the participants in each folic acid (FA) treatment arm were switched to placebo at week 12. All participants received arsenic-removal water filters at baseline to be used for the duration of the 24 week study period and thereafter. **(B)** The first set of analyses for the current study used peripheral blood mononuclear cell (PBMC) samples collected at baseline from a total of 317 FACT participants. We show here the distribution of the 317 participants by treatment arm. By design, the majority of participants in the current study were from the placebo and the 400 μg FA/day treatment arms. To ensure sufficient statistical power for regression analyses, an additional 123 PBMC samples were selected from participants in other treatment arms who had measures for all covariates of interest. All cross-sectional analyses for the current study used PBMC samples that were collected from these 317 participants at baseline, prior to the provision of arsenic-removal water filters and nutritional supplements or a placebo **(C)** The second set of analyses for the current study used all available samples from the placebo group (n = 60). These samples were collected at baseline and at week 12. Participants in the placebo group did not receive any nutritional interventions during the study period but, like all other FACT participants, received arsenic-removal water filters at baseline to be used during the study period and thereafter.
